# Predicting resistance to neoadjuvant chemotherapy in osteosarcoma using machine learning with clinical data and T2-weighted MRI radiomics

**DOI:** 10.1186/s41747-026-00732-z

**Published:** 2026-05-20

**Authors:** Papangkorn Inkeaw, Dumnoensun Pruksakorn, Salita Angkurawaranon, Wipawee Morakote, Pattira Boonsri, Rattanathon Phettom, Thanat Kanthawang

**Affiliations:** 1 Data Science Research Center, Faculty of Science, Chiang Mai University, Chiang Mai, Thailand; 2 Global Health and Chronic Conditions Research Group, Faculty of Medicine, Chiang Mai University, Chiang Mai, Thailand; 3 Department of Computer Science, Faculty of Science, Chiang Mai University, Chiang Mai, Thailand; 4 Department of Orthopedics, Faculty of Medicine, Chiang Mai University, Chiang Mai, Thailand; 5 Center of Multidisciplinary Technology for Advanced Medicine (CMUTEAM), Faculty of Medicine, Chiang Mai University, Chiang Mai, Thailand; 6 Department of Radiology, Faculty of Medicine, Chiang Mai University, Chiang Mai, Thailand; 7 Department of Radiology, Faculty of Medicine, Prince of Songkla University, Songkhla, Thailand

**Keywords:** Artificial intelligence, Drug resistance (neoplasm), Magnetic resonance imaging, Osteosarcoma, Radiomics

## Abstract

**Objectives:**

Identifying patients at risk of chemoresistant osteosarcoma enables risk-adapted management. This study aimed to predict chemoresistant osteosarcoma using baseline clinical and magnetic resonance (MRI)-derived radiomics features, with histological response as the reference standard and external validation included.

**Materials and methods:**

This retrospective single-center study included 115 patients with osteosarcoma from an institutional registry as the internal cohort, divided into training and test sets, and 49 patients from another institution as an external validation cohort. Tumor and peritumoral regions were manually segmented on baseline fat-suppressed T2-weighted MRI. Radiomics features were extracted using PyRadiomics, followed by two feature selection methods to identify potential predictors. Six machine learning models with varying feature combinations were trained to classify histologic chemoresistance in the internal training set. Model performance was assessed in the internal test set, and the best model was externally validated.

**Results:**

The support vector machine model combining eight tumor radiomics features and four clinical-imaging parameters (presence of tumor necrosis > 50% on contrast-enhanced MRI, age, body mass index, and presence of metastasis at presentation) demonstrated the best performance. In the internal test set, it achieved a sensitivity of 83.3%, a specificity of 72.7%, an area under the receiver operating characteristic curve (AUROC) of 0.84, and a positive likelihood ratio of 3.06. External validation yielded a sensitivity of 88.5%, a specificity of 47.8%, and an AUROC of 0.77.

**Conclusion:**

A model combining tumor radiomics and clinical parameters at diagnosis showed strong performance in predicting chemoresistant osteosarcoma, with results confirmed by external validation. This approach may support personalized treatment strategies in high-grade osteosarcoma.

**Relevance statement:**

The validated model may support early, individualized osteosarcoma management.

**Key Points:**

- Baseline T2-weighted MRI radiomics and clinical data can predict chemoresistant osteosarcoma.
- Tumor radiomics combined with clinical features achieved strong predictive accuracy.
- The support vector machine model reached an AUROC of 0.84 for the internal testing and of 0.77 for the external validation.
- The validated model may support early, individualized osteosarcoma management.

**Graphical Abstract:**

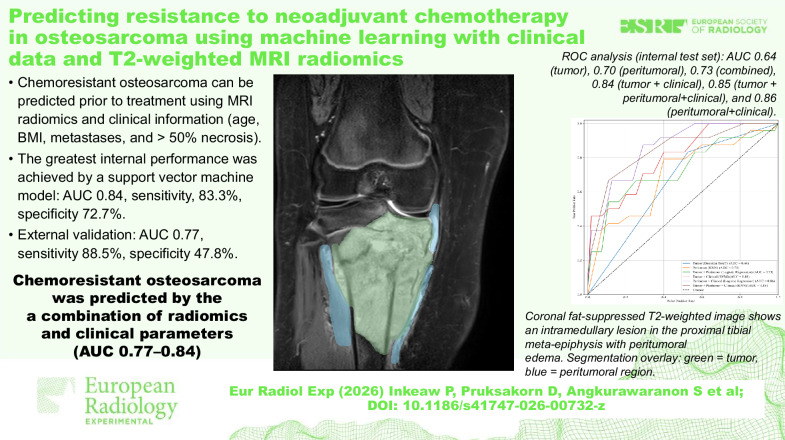

## Background

Osteosarcoma is the most common primary malignant bone tumor [1, 2]. Neoadjuvant chemotherapy combined with surgery has improved survival, yet nearly half of patients respond poorly, limiting limb-salvage options and necessitating more aggressive treatment [3, 4]. Histological assessment of tumor necrosis is the gold standard for evaluating chemotherapy response, but it is only available postoperatively, restricting its role in early treatment planning [3].

Magnetic resonance imaging (MRI) is routinely used for staging and surgical planning, though conventional MRI has limited accuracy in predicting chemotherapy response [5, 6]. Although advanced MRI techniques—such as diffusion-weighted imaging and dynamic contrast-enhanced studies—have shown promise, conventional MRI remains the most widely available modality in routine clinical practice [7, 8]. Studies evaluating baseline MRI predictors of histological response are limited and yield inconsistent results [5, 6], even when combined with clinical or genomic markers [9, 10]. Currently, no validated biomarkers exist for predicting chemoresponsiveness in osteosarcoma [7, 11]. However, baseline tumor characteristics may reflect biological aggressiveness and intratumoral heterogeneity associated with chemoresistance.

Radiomics enables the extraction of quantitative features from routine imaging and, combined with machine learning, has emerged as a potential prognostic tool [10, 12–14]. Prior studies in osteosarcoma were encouraging but lacked external validation and rarely assessed peritumoral features [15]. Early identification of patients at risk for poor chemotherapy response could facilitate risk-adapted management, including closer surveillance or earlier surgical intervention, thereby improving outcomes in high-grade osteosarcoma [16, 17].

Therefore, this study aimed to develop and externally validate an MRI-based radiomics model to predict chemoresistant osteosarcoma using histological response as the reference standard.

## Methods

**Fig. 1:**
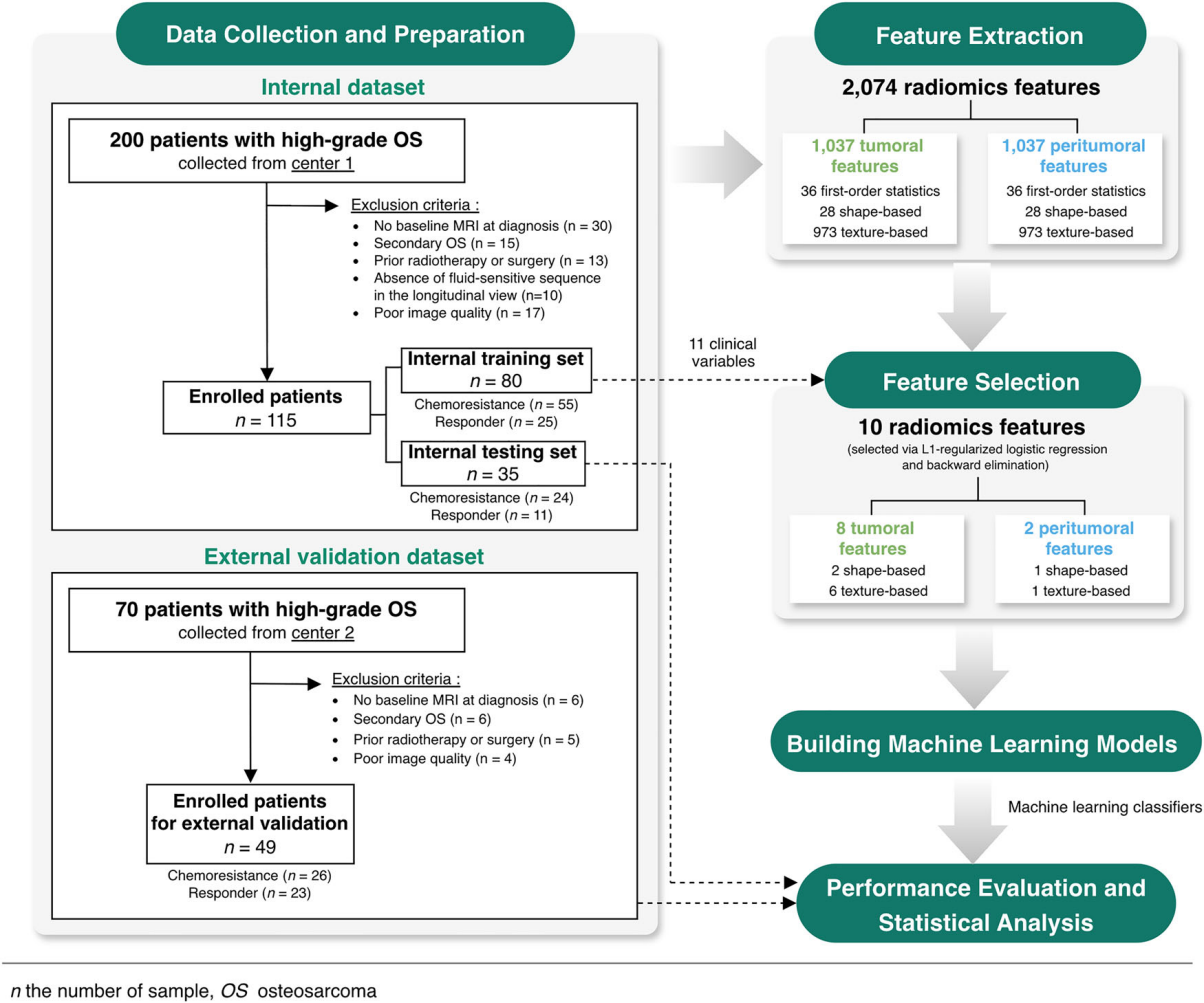
Framework diagram of this study

As shown in Fig. 1, the workflow of this study is structured into the following steps: (1) data collection and preparation; (3) feature extraction; (4) feature selection; (5) building machine learning models; and (6) performance evaluation and statistical analysis. This study was conducted in accordance with the CheckList for EvaluAtion of Radiomics research (CLEAR) checklist [18]. The explanation of each step is given as follows.

### Data collection and preparation

In this study, we acquired two datasets, *i.e*., an internal set and an external validation set. For the internal set, a retrospective query of all high-grade osteosarcoma (Enneking staging IIB–III) in the cancer registry of the Faculty of Medicine, Chiang Mai University, from January 2008 to September 2024 was performed, yielding 200 patients. Patients without baseline MRI at the time of diagnosis (*n* = 30) were excluded. Additional exclusion criteria included secondary osteosarcoma (*n* = 15), prior treatment with radiotherapy or surgery (*n* = 13), absence of fluid-sensitive sequences in the longitudinal view (*n* = 10), and poor image quality (*n* = 17). Finally, 115 patients were included in the internal dataset. We randomly divided the internal set into two datasets, namely the internal training and internal test datasets. The sample ratio between the internal training and internal test datasets was 70:30. The internal training dataset includes 80 patients used for training machine learning models. The remaining 35 patients were included in the internal test dataset for evaluating the performance of the models. There was no overlap of patients between the two datasets.

For the external validation set, we retrospectively included patients from the Faculty of Medicine, Prince of Songkla University. All patients had a diagnosis of high-grade osteosarcoma (Enneking stage IIB–III [19]) at a university-affiliated tertiary care center (Prince of Songkla University, Songkla, Thailand) between January 2015 and September 2024, yielding 70 patients. Patients without baseline MRI at the time of diagnosis (*n* = 6) were excluded. Additional exclusion criteria included post-treatment with radiotherapy or surgery prior to imaging (*n* = 5), secondary osteosarcoma (*n* = 6), and poor image quality (*n* = 4), resulting in the exclusion of 15 cases. Finally, 49 patients were included in this study.

All patients in both datasets received neoadjuvant chemotherapy followed by surgery, with limb salvage when feasible. Regimens included doxorubicin–cisplatin for patients ≥ 15 years and carboplatin–doxorubicin for those < 15 years, administered pre- and postoperatively. Since 2014, high-dose methotrexate (12 g/m^2^/day) has been added to the treatment protocol for pediatric patients.

In the internal dataset, most MRI examinations (85/115; 73.9%) were performed at our institution using GE Healthcare scanners, primarily 1.5-T systems (Signa Excite HD or HDxt), with 15 patients (15/115; 13.0%) scanned on a 3-T system (Signa Pioneer). The remaining patients (30/115; 26.09%) underwent MRI at external centers, mainly on 1.5-T Siemens scanners (19 examinations used Magnetom, Siemens Healthineers). Detailed acquisition parameters are provided in Supplementary Tables S1–S3. In the external validation cohort, most studies (40/49; 81.6%) were acquired on a 1.5-T Philips Ingenia scanner, with the remainder performed at referring centers (Supplementary Table S4).

MRI protocols included T1-weighted and fluid-sensitive sequences—fat-suppressed T2-weighted or short tau inversion recovery (STIR)—obtained in the axial plane and at least one orthogonal plane. Most internal studies used fat-suppressed T2-weighted imaging, while 19/115 cases (16.5%) used STIR; all external cases used fat-suppressed T2-weighted. Slice thickness ranged from 4.0–6.5 mm (internal) and 3.0–4.0 mm (external), with in-plane resolutions of 160–512 × 256–380 pixels and 159–308 × 188–512 pixels, respectively. Contrast-enhanced fat-suppressed T1-weighted images were acquired in multiple planes, and T1-weighted sequences were used to assess skip metastases.

**Fig. 2:**
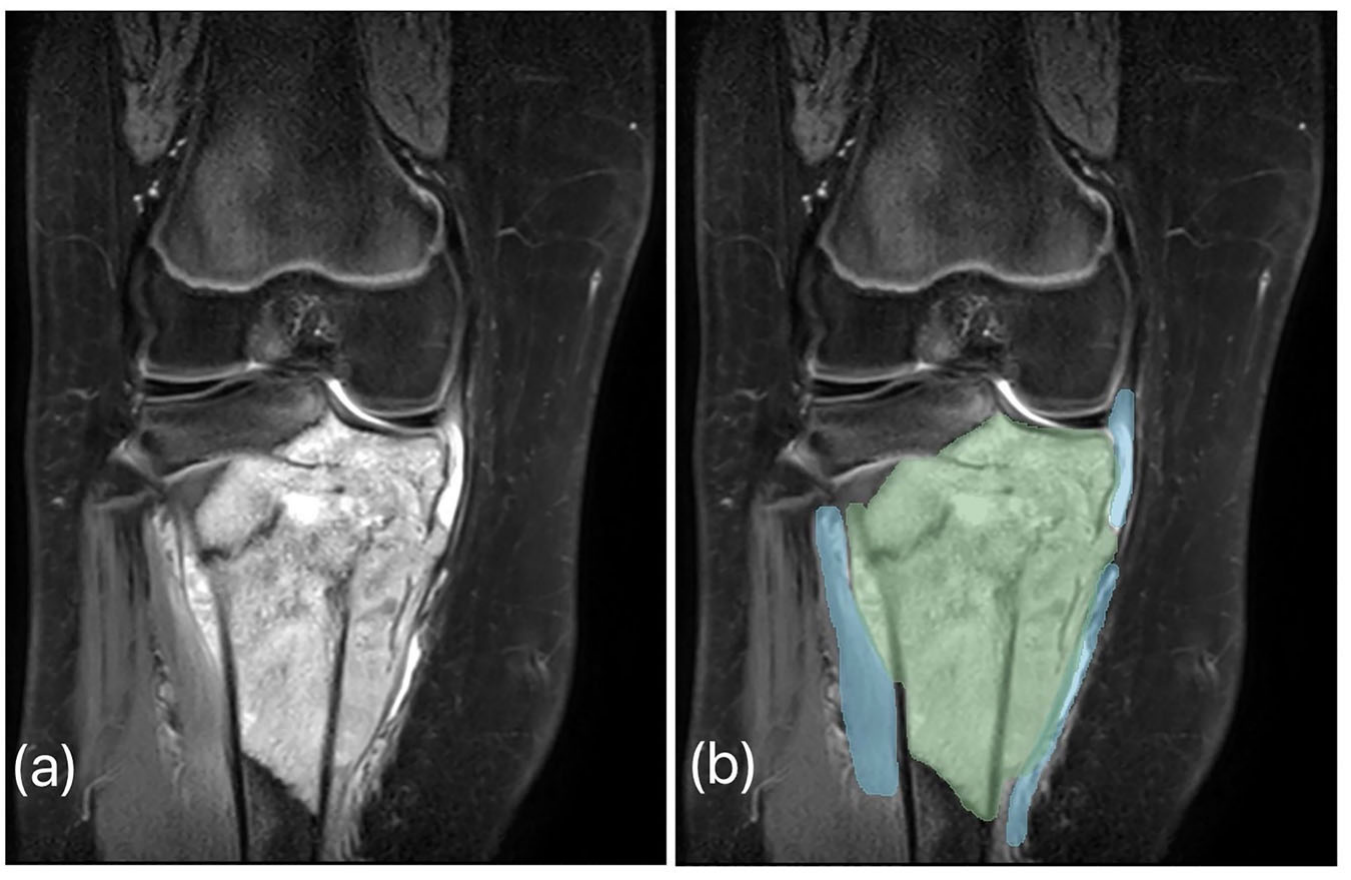
**a** Coronal fat-suppressed T2-weighted image shows an intramedullary lesion in meta-epiphysis of the proximal tibia with associated peritumoral soft tissue edema. **b** Tumor segmentation overlay on the original image, with the green area representing the tumor and the blue area indicating the peritumoral region

We assessed radiomics analysis of fat-suppressed T2-weighted images or STIR in the predictive assessment of histologic necrosis because this sequence is a core constituent of clinical MRI examinations of musculoskeletal tumors and is sensitive to tumor water distribution, tumor extent, as well as perilesional tumor-related marrow and soft tissue edema [20, 21]. The radiomics features of the tumor and peritumoral regions have been considered. The two regions have been manually annotated by board-certified radiologists blinded to clinical information using the semiautomated Synapse 3D software on longitudinal images and were subsequently validated by a musculoskeletal radiologist (T.K.) with 11 years of experience (Fig. 2). In cases of segmentation disagreement, discrepancies were resolved by consensus among all radiologist co-authors.

Additionally, clinical-imaging parameters, including age, body mass index (BMI), gender, Enneking staging [22], initial metastasis, central necrosis > 50%, pathologic subtype, tumor location, tumor volume, peritumoral volume, tumor volume per BMI, and peritumoral volume per BMI, were collected and used in this study. Tumor necrosis was visually categorized as < 50% or ≥ 50% of the entire tumor demonstrating non-enhancement on contrast-enhanced fat-suppressed T1-weighted imaging, in accordance with previous literature [23]. Tumor and peritumoral volumes were calculated from the segmented tumor and peritumoral areas using the Synapse 3D software [24]. Furthermore, the percentage of tumor necrosis was collected from the histological report and considered as a patient’s response to chemotherapy, which is the outcome in this study. The tumor necrosis ≥ 90% was defined as a histologic responder. Otherwise, the necrosis < 90% was a histologic resistance, in accordance with the widely accepted cutoff in the literature [25]. Histologic responses to preoperative chemotherapy in resected specimens were reviewed by a musculoskeletal pathologist at each institution.

### Feature extraction

The radiomics features in the tumor and peritumoral regions were extracted separately. They consist of three feature categories: (1) first-order statistics features; (2) shape-based features; and (3) textural features. First-order features quantify the distribution of voxel intensities within regions of interest, providing basic statistical information. Shape-based features capture the geometric properties of the regions of interest, such as size, surface, and compactness. Textural features describe the spatial arrangement and relationship of gray-level intensities, characterizing internal heterogeneity within the regions. Texture quantification was performed using several established methods: gray-level co-occurrence matrix (GLCM) [26]; gray-level dependence matrix (GLDM) [27]; gray-level run-length matrix (GLRLM) [28]; gray-level size-zone matrix (GLSZM) [29]; and neighborhood gray-tone difference matrix (NGTDM) [30]. To enhance the visibility of structural details, such as edges, patterns, and textures, a Laplacian of Gaussian (LoG) filter and a wavelet decomposition were applied to the tumor and peritumoral regions. Textural features were subsequently extracted from both the original and the filtered images to capture multiscale and frequency-based information.

For each region, 1,037 features, including 36 first-order statistics features, 28 shape-based features, and 973 textural features, were extracted using the PyRadiomics library version 3.1.0 [31, 32]. All radiomics feature extraction parameters were configured using the default settings of the library, following the specifications provided in the official PyRadiomics documentation (https://pyradiomics.readthedocs.io/en/latest/index.html). Before extracting features, the intensity levels within a region of interest were discretized using a fixed bin width of 25 intensity units. Additionally, 11 clinical-imaging parameters were included, resulting in a total of 2,085 features for a patient.

### Feature selection

In this step, we identified the radiomics features that have the potential to predict the outcome. Two feature selection methods were employed sequentially. First, a logistic regression with L1 regularization was used to predict the outcome (dependent variable) using all features as predictors (independent variables). The features were then ranked by the absolute values of their coefficient in the regression model. A feature with a high absolute coefficient value indicates a higher effect on the prediction outcome than one with a lower coefficient value. In this study, we selected the features whose absolute coefficient value is higher than the mean of the absolute coefficient values. Consequently, 49 features were selected. Next, the selected features were refined by the backward elimination method. Starting with the 49 features, the method removed the least significant features one by one from a model (*i.e*., in this study, a random forest model) until the number of selected features was less than or equal to 10. Finally, the selected 10 key features comprised eight radiomics features from the tumor region and two from the peritumoral regions.

Baseline clinical-imaging parameters at diagnosis included age, BMI (calculated as weight in kilograms divided by height in meters squared), gender, presence of initial metastasis, Enneking stage, tumor location, and presence of > 50% tumor necrosis on baseline post-contrast MRI, tumor volume, peritumoral volume, tumor volume adjusted for BMI, and peritumoral volume adjusted for BMI. The Enneking staging system classifies bone tumors based on grade, location, and metastatic potential: Stage IIA denotes high-grade, intracompartmental tumors; Stage IIB refers to high-grade, extracompartmental tumors; and Stage III includes any grade with distant metastasis. Tumor and peritumoral volume were calculated using the segmented area obtained from feature extraction. The extent of necrosis was visually categorized as ≤ 50% or > 50% of the total tumor area. Finally, four clinical-imaging parameters were selected for further analysis based on statistical significance (*p* < 0.05) and supporting literature [13, 23], including age, BMI, presence of initial metastasis, and > 50% tumor necrosis on post-contrast MRI.

We now have three feature groups, comprising clinical-imaging parameters, radiomics features from the tumor region, and features from the peritumoral region. Six feature sets were investigated in our experiment. Radiomics features from the tumor and peritumoral regions were individually examined. Combining features from the tumor and peritumoral regions was also considered. Remaining, we merged clinical-imaging parameters into each of the first three feature sets. The features were standardized using *z*-score normalization to ensure comparable feature scales and were subsequently fed to classification models as input to predict the outcome.

### Building machine learning models

Five machine learning models, decision tree, logistic regression, random forest, support vector machine (SVM), and k-nearest neighbor (kNN), were evaluated to represent a range of complementary learning paradigms commonly applied in radiomics studies. All classifiers were trained using the internal training dataset, and their hyperparameters were optimized using grid search cross-validation.

Logistic regression was used as an interpretable linear baseline model frequently applied in clinical research. Decision tree was included as an interpretable nonlinear model capable of capturing simple decision rules. Random forest, an ensemble extension of decision trees, was employed to model complex feature interactions and reduce overfitting. SVM was selected for its effectiveness in high-dimensional, limited-sample-size settings characteristic of radiomics data. kNN was incorporated as a non-parametric, instance-based classifier without assumptions regarding data distribution.

All models were evaluated under the same preprocessing, feature selection, and validation framework to ensure fair comparison. To address class imbalance, class weights were assigned inversely proportional to class frequencies in the training dataset, giving greater weight to the minority class (responders) and lower weight to the majority class (chemoresistant cases), thereby balancing their contribution to the loss function.

### Performance evaluation and statistical analysis

Statistical analyses were performed using Python (version 3.7.0) with a two-sided significance level of 0.05. Sample size was estimated using G*Power based on a multivariate analysis of variance (MANOVA) framework to predict histologic response (good *versus* poor responders). Assuming seven predictor variables, 5% significance, 95% power, and a large effect size, the required sample size was 164 participants. The cohort was accordingly divided into 115 patients (70%) for model development and 49 patients (30%) for external validation.

The significant differences in demographic and clinical parameters between outcomes were compared in both the internal set and the external validation set. Continuous variables, including age, BMI, tumor volume, peritumoral volume, tumor volume per BMI, and peritumoral volume per BMI, were analyzed using the Wilcoxon rank-sum (Mann–Whitney) test. Meanwhile, categorical variables, including gender, Enneking stage, initial metastasis, central necrosis > 50%, pathology, and tumor location, were analyzed using Fisher’s exact test. Subgroup analyses were performed after excluding patients imaged with STIR sequences or 3-T scanners.

The internal test dataset was used to evaluate the performance of the machine learning models. The sensitivity, specificity, positive likelihood ratio and area under the receiver operating characteristic curve (AUROC) were used as classification performance matrices. The best model was identified based on the classification performance. DeLong’s test was applied to evaluate statistically significant differences between the AUROCs of each model. It was then validated using the external validation set to assess its generalization.

## Results

Of 115 patients, 79 (68.7%) had histologic chemoresistance (estimated incidence: 60.1%). The median age was 16 years, with a balanced sex distribution (47.0% female). Notably, 58 patients (50.4%) received carboplatin and doxorubicin, with or without high-dose methotrexate, due to being younger than 15 years of age. Most had conventional osteosarcoma (81.7%), and 21.7% had metastasis (Enneking stage III). Chemoresistant patients more frequently had chondroblastic (12.7%) or telangiectatic (8.9%) subtypes, which were absent in responders (*p* = 0.025). Metastasis was more common in the chemoresistant group (35.4% *versus* 16.7%; *p* < 0.001), and their BMI was lower (17.84 *versus* 19.76; *p* = 0.013). Age, gender, tumor location, volume, and peritumoral volume showed no significant differences among two patient groups.

Specifically, patients with chemoresistance showed greater tumor necrosis (> 50%) in both the internal training set (31/55; 56.4%) and internal testing set (15/24; 62.5%). Table 1 summarizes the clinical and imaging parameters for both the internal training and testing datasets. In the internal testing dataset, chemoresistant patients were more likely to be older (*p* = 0.014), female (*p* = 0.027), and have tumors in axial locations (*p* = 0.036). No statistically significant differences were found in the remaining clinical or imaging parameters between the chemoresistance and responder groups. The results remained significant in subgroup analyses after excluding patients imaged with STIR sequences or 3-T scanners. Based on statistically significant findings in the internal training dataset and literature review [23], we selected the following variables for clinical-imaging parameters in the model, including the presence of tumor necrosis > 50%, age, BMI, and the presence of initial metastasis.

**Table 1:** Clinical and imaging parameters of internal training and internal testing datasets

Clinical and imaging parameters	Internal training dataset (*n* = 80)			Internal testing dataset (*n* = 35)		
	Chemoresistance (*n* = 55)	Responder (*n* = 25)	*p*-value**	Chemoresistance (*n* = 24)	Responder (*n* = 11)	*p*-value**
Age (years)*	16.0 (14.0‒21.5)	15.0 (11.0‒19.0)	0.144	16.5 (12.8‒36.5)	12.0 (11.5‒14.0)	**0.014**
BMI (kg/m^2^)*	19.2 (17.3‒21.9)	17.8 (17.0‒20.4)	0.172	20.5 (15.5‒23.0)	17.1 (14.6‒19.4)	0.088
Female gender	29 (52.73%)	12 (48.00%)	0.810	12 (50.00%)	1 (9.09%)	**0.027**
Initial metastasis	18 (32.73%)	5 (20.00%)	0.295	10 (41.67%)	2 (18.18%)	0.259
Enneking staging						
IIA	26 (47.27%)	17 (68.00%)	0.210	7 (29.17%)	7 (63.64%)	0.154
IIB	17 (30.91%)	4 (16.00%)		9 (37.50%)	2 (18.18%)	
III	12 (21.82%)	4 (16.00%)		8 (33.33%)	2 (18.18%)	
Location						
Axial skeleton	8 (14.55%)	1 (4.00%)	0.302	5 (20.83%)	0 (0.00%)	**0.036**
Upper extremity	4 (7.27%)	1 (4.00%)		0 (0.00%)	2 (18.18%)	
Lower extremity	43 (78.18%)	23 (92.00%)		19 (79.17%)	9 (81.82%)	
Presence of tumor necrosis > 50%	31 (56.36%)	7 (28.00%)	**0.029**	15 (62.50%)	1 (9.09%)	**0.000**
Tumor volume (mL)*	302.0 (163.4‒568.4)	277.5 (89.2‒371.7)	0.132	348.5 (115.5‒693.8)	151.1 (85.7‒329.4)	0.234
Peritumoral volume (mL)*	246.6 (107.6‒397.4)	155.1 (40.0‒270.5)	0.078	160.7 (106.9‒274.2)	112.8 (53.5‒134.6)	0.106
Tumor volume per BMI (mL·m^2^/kg)*	15.6 (9.6‒35.0)	13.9 (4.6‒24.7)	0.202	18.9 (5.8‒27.9)	10.3 (5.6‒18.6)	0.346
Peritumoral volume per BMI (mL·m^2^/kg)*	12.8 (5.9‒19.4)	9.0 (2.1‒16.7)	0.177	7.6 (4.9‒16.1)	6.1 (2.6‒8.6)	0.365

* Median (25th‒75th percentiles)

** *p*-values lower than 0.05 were considered statistically significant. (Values in bold indicate statistically significant *p*-values.)

In the external validation cohort (*n* = 49), 26 patients (53.1%) exhibited histologic chemoresistance. The median age was 13 years (range 9–70), and 31 patients (63.3%) received carboplatin and doxorubicin, with or without high-dose methotrexate, owing to age < 15 years. The cohort had a near-equal sex distribution (34.7% female). Conventional osteosarcoma was the predominant histologic subtype (41/49; 83.7%), and 16 patients (32.7%) presented with regional or distant metastasis (Enneking stage III). Patients with chemoresistance had a significantly higher BMI compared to responders (20.4 *versus* 17.19 kg/m²; *p* = 0.011). No other clinical or imaging parameters showed statistically significant differences between the two groups (Table 2).

**Table 2:** Clinical and imaging parameters of the external validation dataset

Clinical and imaging parameters	Response to chemotherapy of the external dataset (*n* = 49)		
	Chemoresistance (*n* = 26)	Responder (*n* = 23)	*p*-value^**^
Age (years)*	13.5 (12.0‒19.8)	13.0 (12.0‒16.5)	0.665
BMI (kg/m^2^)*	20.4 (17.7‒24.6)	17.2 (14.5‒20.0)	**0.011**
Female gender	8 (30.77%)	9 (39.13%)	0.564
Initial metastasis	10 (38.46%)	6 (26.09%)	0.382
Enneking staging			
IIA	6 (23.08%)	8 (34.78%)	0.560
IIB	10 (38.46%)	9 (39.13%)	
III	10 (38.46%)	6 (26.09%)	
Location			
Axial skeleton	3 (11.54%)	0 (0.00%)	0.243
Upper extremity	3 (11.54%)	3 (13.04%)	
Lower extremity	20 (76.92%)	20 (86.96%)	
Presence of tumor necrosis > 50%	11 (42.31%)	6 (26.09%)	0.367
Tumor volume (mL)*	240.9 (81.6‒599.2)	269.6 (157.7‒539.3)	0.465
Peritumoral volume (mL)*	158.4 (36.3‒288.5)	155.1 (113.3‒388.7)	0.496
Tumor volume per BMI (mL·m^2^/kg)*	11.1 (3.8‒31.3)	16.8 (10.9‒27.9)	0.275
Peritumoral volume per BMI (mL·m^2^/kg)*	7.8 (2.0‒13.0)	9.2 (6.5‒18.2)	0.158

* Median (25th‒75th % percentiles)

** *p*-values lower than 0.05 were considered statistically significant. (Values in bold indicate statistically significant *p*-values.)

**Fig. 3:**
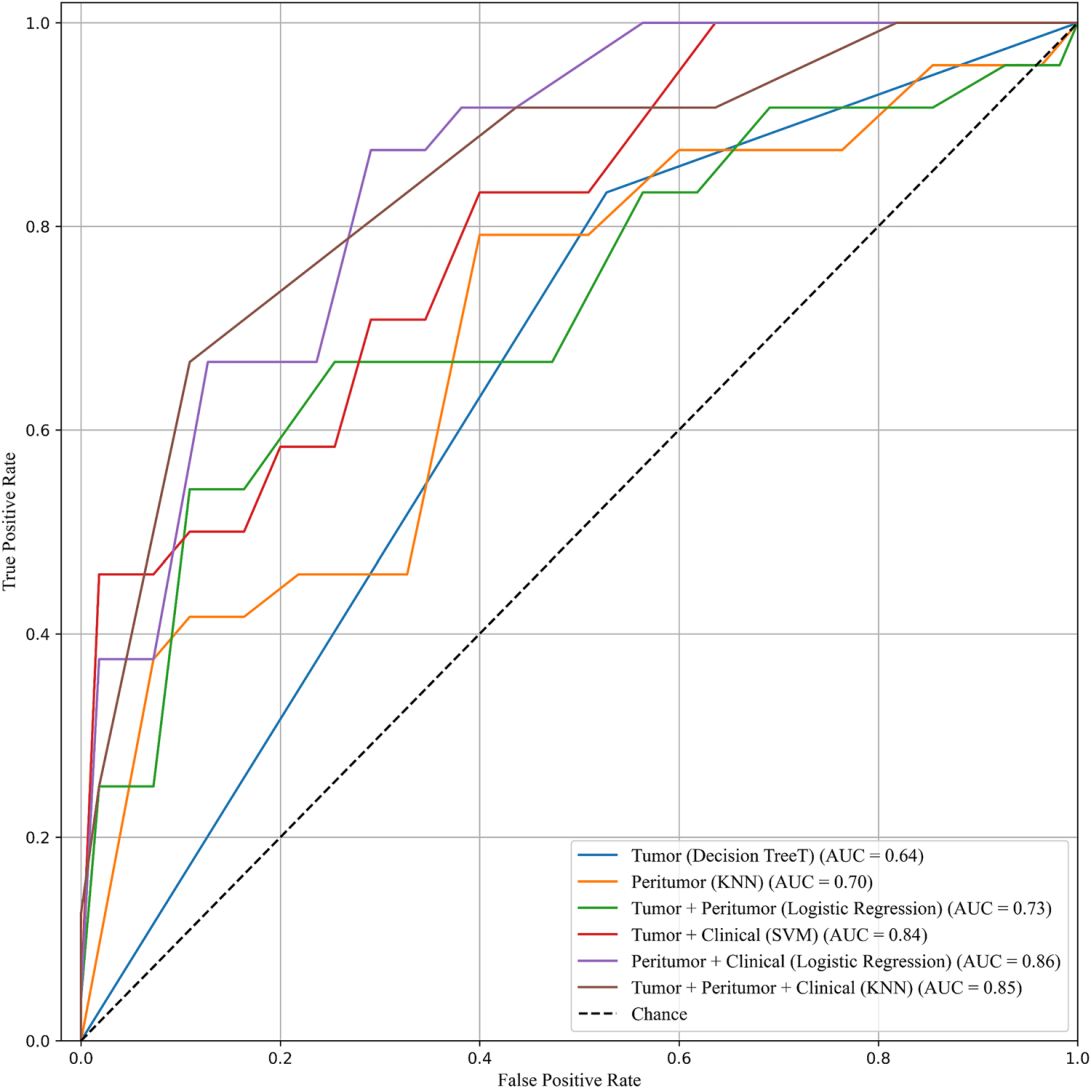
Performance of clinical and radiomics models in predicting histologic chemoresistance in the internal test dataset. The area under the receiver operating characteristic curve (AUROC) was 0.64 for tumor radiomics, 0.70 for peritumoral radiomics, and 0.73 for combined tumor + peritumoral radiomics. Performance improved with the addition of clinical-imaging factors, reaching AUROCs of 0.84 (tumor + clinical-imaging), 0.85 (tumor + peritumoral + clinical-imaging), and 0.86 (peritumoral + clinical-imaging)

From the feature selection step, the top 20 radiomics features ranked by their weight obtained from a logistic regression model with L1 regularization were provided in Supplementary Material Table S5. As given in Table 3, ten radiomics features were chosen from the feature selection step. Eight of the selected features were extracted from the tumor region. They are two shape-based, three GLRLM, two GLCM, and one GLDM features. Meanwhile, two features from the peritumoral region were selected, including the maximum diameter and one GLDM feature. In addition, features were ranked using permutation feature importance scores [33] derived from the best-performing SVM with an RBF kernel. This model primarily relied on tumor shape features, followed by gray-level dependence features from both tumor and peritumoral regions and gray-level run-length features from the tumor. Tumor volume showed the smallest positive importance, while peritumoral diameter and tumor gray-level co-occurrence features had negative importance scores, suggesting limited contribution despite selection. Permutation importance was used solely to interpret the trained SVM model; although model-agnostic, importance scores remain dependent on the specific trained model and may vary with different algorithms. The predictive performance of each model is summarized in Table 4. Models using tumor or peritumoral radiomics alone showed modest discrimination, with AUROCs ranging from 0.50 to 0.70. Logistic regression models using either tumor or peritumoral radiomics achieved the highest sensitivity (100%), while the SVM model using peritumoral features showed the highest specificity (54.5%). Combining radiomics features with clinical-imaging parameters improved model performance (Table 4 and Fig. 3). Tumor radiomics alone demonstrated modest discriminatory performance (AUROC = 0.64; decision tree model), which improved substantially when combined with clinical-imaging factors (AUROC = 0.84; SVM; *p* = 0.013) or with both peritumoral radiomics and clinical-imaging factors (AUROC = 0.85; kNN; *p* = 0.004). The highest overall performance was achieved by the model combining peritumoral radiomics with clinical-imaging parameters (AUROC = 0.86; logistic regression; *p* = 0.001). In contrast, adding peritumoral radiomics to tumor radiomics alone did not significantly improve discrimination (AUROC = 0.73; logistic regression; *p* = 0.074), and the remaining pairwise comparisons were not statistically significant. The highest sensitivity (91.7%) was achieved by the kNN model using all three feature sets, though with moderate specificity (54.5%). The tumor and clinical-imaging SVM model yielded the highest positive likelihood ratio (3.06; 95% CI = 1.15–8.15).

**Table 3:** The radiomics features used for model training, ordered by permutation feature importance scores obtained from an SVM with an RBF kernel

Type	Features	Feature importance
Tumor	Minor longest axis length calculated from the original shape. It can be computed by Minor longest axis = $4\sqrt{{\lambda }_{{minor}}}$, where ${\lambda }_{{minor}}$ is the second-largest axis length of the principal component [15, 49].	0.0077
Peritumoral	Large dependence low gray-level emphasis calculated from the gray-level dependence matrix of the original image [50].	0.0074
Tumor	Dependence of non-uniformity calculated from the gray-level dependence matrix of high pass, high pass, high pass of x, y, z axes of wavelet transforms [27, 51].	0.0038
Tumor	Run length of non-uniformity calculated from the gray-level run length matrix of the original image [27].	0.0036
Tumor	Run length of non-uniformity calculated from the gray-level run length matrix of low pass, low pass, high pass of x, y, z axes of wavelet transforms [28].	0.0026
Tumor	Run length of non-uniformity calculated from the gray-level run length matrix of low pass, low pass, low pass of x, y, z axes of wavelet transforms [28, 51].	0.0026
Tumor	Mesh volume calculated from original shape [51].	0.0015
Peritumoral	Maximum 2D diameter column calculated from original shape [50].	-0.0051
Tumor	Inverse difference normalized calculated from the gray-level co-occurrence matrix of high pass, low pass, high pass of x, y, z axes of wavelet transforms [26, 50].	-0.0073
Tumor	Inverse difference moment normalized calculated from the gray-level co-occurrence matrix of high pass, low pass, high pass of x, y, z axes of wavelet transforms [26, 51].	-0.0129

A run length was defined as a sequence of neighboring pixels with the same intensity in a specific direction, such as left to right, top to bottom, or diagonally

**Table 4:** Predictive values of each model for chemoresistance from the test set (internal validation)

Feature designs	Models	Sensitivity (%) (95% CI)	Specificity (%) (95% CI)	LR + (95% CI)	AUROC (95% CI)
Tumor	Decision tree	83.30 (68.40‒98.20)	45.50 (16.00‒74.90)	1.53 (0.87‒2.70)	**0.64 (0.45‒0.84)**
	Logistic regression	**100.00 (100.00‒100.00)**	0.00 (0.00‒0.00)	1.00 (1.00‒1.00)	0.50 (0.29‒0.71)
	Random forest	91.70 (80.60‒102.70)	27.30 (1.00‒53.60)	1.26 (0.86‒1.85)	0.61 (0.41‒0.81)
	SVM	83.30 (68.4‒98.20)	54.50 (25.10‒84.00)	1.83 (0.94‒3.59)	0.60 (0.40‒0.80)
	kNN	95.80 (87.8‒103.80)	18.20 (0.00‒41.00)	1.17 (0.88‒1.57)	**0.64 (0.41‒0.81)**
Peritumoral	Decision tree	66.70 (47.80‒85.50)	9.10 (0.00‒26.10)	0.73 (0.52‒1.03)	0.50 (0.29‒0.71)
	Logistic regression	**100.00 (100.00‒100.00)**	0.00 (0.00‒0.00)	1.00 (1.00‒1.00)	**0.70 (0.52‒0.88)**
	Random forest	70.80 (52.00‒89.00)	36.40 (7.90‒64.80)	1.11 (0.67‒1.86)	0.53 (0.32‒0.74)
	SVM	70.80 (52.60‒89.00)	54.50 (25.10‒84.00)	1.56 (0.78‒3.13)	0.66 (0.47‒0.84)
	kNN	83.30 (68.40‒98.20)	45.50 (16.00‒74.90)	1.53 (0.87‒2.70)	**0.70 (0.52‒0.88)**
Tumor + peritumoral	Decision tree	54.20 (34.20‒74.10)	36.40 (7.90‒64.80)	0.85 (0.48‒1.52)	0.40 (0.19‒0.61)
	Logistic regression	70.80 (52.60‒89.00)	45.5 (16.00‒74.90)	1.30 (0.72‒2.36)	**0.73 (0.56‒0.90)**
	Random forest	79.20 (62.90‒95.40)	36.40 (7.90‒64.80)	1.24 (0.76‒2.03)	0.63 (0.43‒0.82)
	SVM	50.00 (30.00‒70.00)	54.50 (25.10‒84.00)	1.10 (0.51‒2.36)	0.57 (0.37‒0.77)
	kNN	66.70 (47.80‒85.50)	54.50 (25.10‒84.00)	1.47 (0.72‒2.97)	0.61 (0.41‒0.80)
Tumor + clinical*	Decision tree	83.30 (68.40‒98.20)	36.40 (7.90‒64.80)	1.31 (0.81‒2.12)	0.64 (0.45‒0.83)
	Logistic regression	91.70 (80.60‒102.70)	45.50 (16.00‒74.90)	1.68 (0.97‒2.92)	0.82 (0.68‒0.96)
	Random forest	83.30 (68.40‒98.20)	45.50 (16.00‒74.90)	1.53 (0.87‒2.70)	0.67 (0.49‒0.86)
	SVM	83.30 (68.40‒98.20)	**72.70 (46.40‒99.00)**	**3.06 (1.15‒8.15)**	**0.84 (0.71‒0.97)**
	kNN	87.50 (74.30‒100.70)	54.50 (25.10‒84.00)	1.93 (0.99‒3.74)	0.81 (0.67‒0.95)
Peritumoral + clinical	Decision tree	75.00 (57.70‒92.30)	9.10 (0.00‒26.10)	0.83 (0.61‒1.11)	0.63 (0.43‒0.82)
	Logistic regression	70.80 (52.60‒89.00)	**72.70 (46.40‒99.00)**	2.60 (0.96‒7.05)	**0.86 (0.74‒0.98)**
	Random forest	79.20 (62.90‒95.40)	54.50 (25.10‒84.00)	1.74 (0.88‒3.44)	0.65 (0.46‒0.84)
	SVM	70.80 (52.60‒89.00)	72.70 (46.40‒99.00)	2.60 (0.96‒7.05)	0.81 (0.66‒0.95)
	kNN	87.5 (74.30‒100.70)	45.50 (16.00‒74.90)	1.60 (0.92‒2.81)	0.79 (0.64‒0.94)
Tumor + peritumoral + clinical*	Decision tree	83.30 (68.40‒98.20)	36.40 (7.90‒64.80)	1.31 (0.81‒2.12)	0.64 (0.44‒0.83)
	Logistic regression	83.30 (68.40‒98.20)	63.60 (35.20‒92.10)	2.29 (1.03‒5.11)	0.80 (0.66‒0.95)
	Random forest	83.30 (68.40‒98.20)	45.50 (16.00‒74.90)	1.53 (0.87‒2.70)	0.70 (0.52‒0.88)
	SVM	87.50 (74.30‒100.70)	63.60 (35.20‒92.10)	2.41 (1.09‒5.34)	0.83 (0.69‒0.97)
	kNN	91.70 (80.60‒102.70)	54.50 (25.10‒84.00)	2.02 (104‒3.90)	**0.85 (0.72‒0.98)**

*AUROC* Area under the receiver operating characteristic curve, *CI* Confidence interval, *kNN* k-nearest neighbor, *LR* Likelihood ratios, *SVM* Support vector machine

* Clinical parameters include the presence of tumor necrosis > 50% on post-contrast MRI, age, BMI, and presence of initial metastasis

** Values in bold indicate the highest values for each feature design.

We employed an SVM model with an RBF kernel incorporating tumor radiomics and clinical-imaging factors, as it required fewer input features while achieving accuracy comparable to other combined models and maintaining a balanced trade-off between sensitivity and specificity. In the external validation set, this model achieved a sensitivity of 88.5% (76.2%–100.7%), specificity of 47.8% (27.4%–68.2%), and an AUROC of 0.77 (0.64–0.90).

## Discussion

This study developed MRI-based radiomics models to predict histologic chemoresistance in high-grade osteosarcoma using baseline clinical-imaging parameters. The models included tumor, peritumoral, and combined features, with or without clinical-imaging parameters (age, BMI, initial metastasis, and > 50% necrosis on post-contrast MRI). Integrating radiomics with clinical-imaging parameters improved model performance, with internal AUROCs ranging from 0.50 to 0.86. However, adding peritumoral features to tumor radiomics did not significantly enhance predictive accuracy compared to tumor radiomics alone. The model combining tumor radiomics and clinical-imaging parameters using an SVM algorithm achieved an internal AUROC of 0.84, the highest positive likelihood ratio of 3.06, and an external AUROC of 0.77. These findings highlight the potential of radiomics, especially when integrated with clinical-imaging data, for early prediction and personalized treatment in osteosarcoma.

Standard imaging evaluation of suspected osteosarcoma relies on MRI, with computed tomography (CT) reserved for cases in which MRI is contraindicated, primarily for surgical staging and planning. However, baseline conventional MRI with or without contrast and CT have shown limited and inconsistent accuracy for risk stratification and prediction of chemotherapy response. Radiomics across multiple baseline imaging modalities offers a promising approach to improve prediction of histologic response and survival outcomes [10, 13, 15, 34]. Combining pre- and post-chemotherapy radiomics data further improves baseline model accuracy [15] but may delay treatment adjustments, which is a critical consideration for personalized medicine. Although radiomics using baseline advanced MRI techniques, such as diffusion-weighted imaging, may achieve higher predictive performance, their specialized acquisition and limited routine availability restrict clinical use [15]. Post-contrast T1-weighted MRI has been reported to provide slightly higher predictive accuracy for radiomics-based assessment of histologic response in osteosarcoma [35]; however, it requires contrast administration, time consuming and protocol standardization and may be unsuitable for some patients, particularly pediatric populations or those with contraindications. Positron emission tomography (PET-) and CT-based radiomics are further limited by cost, radiation exposure, and the need for additional imaging beyond the standard of care [15, 34]. Native non–contrast-enhanced T1-weighted MRI primarily provides anatomic information with limited sensitivity to tumor heterogeneity. In contrast, fluid-sensitive sequence (T2-weighted or STIR) is universally acquired, effectively depicts tumor heterogeneity and peritumoral edema, and is widely accessible. Focusing on this sequence therefore prioritizes clinical feasibility and generalizability for radiomics-based risk stratification [10, 14].

In addition, prior studies in osteosarcoma mouse models have demonstrated excellent repeatability and reproducibility of quantitative T2-based measurements, supporting the technical robustness of T2-derived features [36]. However, challenges remain. A systematic review by Zhong et al [15]. highlighted several limitations in radiomics studies, including retrospective designs, small sample sizes, limited external validation, and a lack of standardized pipelines. All these factors hinder clinical translation. To date, only two studies have used T2-weighted radiomics to predict histologic chemoresistance in osteosarcoma. White et al used baseline T2-weighted tumor radiomics alone, reporting an AUROC of 0.71 [10], while Zhong et al developed a clinical-radiomics nomogram incorporating T2-weighted features and post-chemotherapy biomarkers (uric acid, white blood cell count, and serum potassium), achieving a higher AUROC of 0.79 [14]. Our study reinforces the value of integrating clinical-imaging and radiomic features, demonstrating slightly higher performance (AUROCs 0.84–0.86 internally, 0.77 externally). Notably, ours is the first to include external validation, supporting its robustness and clinical relevance. Importantly, the model relies solely on baseline clinical-imaging parameters, enabling early identification of chemoresistant patients and thereby facilitating optimized, individualized management, such as closer monitoring with interim MRI, consideration of alternative regimens, or earlier surgical intervention [16, 17].

In our best-performing model (see Table 3), two of the eight selected features were related to tumor size, while the remaining six were texture descriptors, most derived from wavelet-transformed images. Tumor size is not only a marker of intrinsic tumor biology but also reflects metastatic potential and treatment resistance in osteosarcoma [23, 37, 38]. Larger tumors are typically more cellular, harbor a greater proportion of chemoresistant clones, and often demonstrate necrotic areas due to impaired perfusion [3]. Both necrosis and chondroblastic components, which can mimic necrosis on MRI, exhibit reduced vascularity, thereby limiting drug delivery and contributing to chemoresistance [23, 39, 40]. This provides a biological rationale for the predictive value of radiomics features related to tumor size and heterogeneity.

Although a previous study [14] reported the use of shape-based features to predict response to neoadjuvant chemotherapy in osteosarcoma, it incorporated only a single texture feature. In contrast, our results highlight the greater importance of texture features, which outperformed shape features in the best-performing model. This is consistent with another study [10], which demonstrated the utility of texture features in identifying histologic tumor necrosis, with more than half derived from GLCM, GLDM, and GLRLM matrices, similar to those used in our analysis. Moreover, our radiomics-based model achieved superior performance (AUROC = 0.84) compared with an established clinical prediction rule (AUROC = 0.76), which incorporated age > 40 years, baseline metastasis, tumor volume, and pre-chemotherapy necrosis > 50% [9]. By capturing subtle texture patterns across multiple orientations and frequencies, features imperceptible to human observers, radiomics provides added diagnostic and prognostic value beyond conventional imaging. These findings highlight the potential of radiomics as a powerful support tool for guiding individualized treatment strategies.

To the best of our knowledge, this is the first study to evaluate peritumoral radiomics in osteosarcoma. Peritumoral radiomics alone showed a slightly higher AUROC (0.70) than tumor radiomics (0.64), though the difference was not statistically significant. While peritumoral soft tissue edema is a known feature of malignant bone tumors [16, 41–43] and may relate to chemoresistance [23], its underlying pathology in untreated osteosarcoma remains unclear. Histologic analysis of high signal areas on T2-weighted or STIR images after chemotherapy has revealed variable findings, including edema, hemorrhage, fibrosis, necrosis, and viable tumor cells [16, 41–43]. Although combining peritumoral radiomics with clinical-imaging parameters yielded the highest AUROC, it did not significantly outperform models based on tumor radiomics and clinical-imaging parameters. Additionally, combining tumor and peritumoral features added complexity and was more time-consuming without improving performance. Therefore, tumor radiomics may be more practical and efficient for treatment planning.

Our study has both strengths and limitations. In terms of strengths, first, although it was a single-center retrospective study, the completeness of clinical data (collected using standardized record forms in our tumor registry), along with a priori sample size calculation and the inclusion of an independent external validation set from another institution, helps mitigate potential bias and overfitting on the model. Notably, no missing data were present in this study. Second, this model was derived using the histology from surgical specimens, which is the gold standard for evaluating chemoresponsiveness and is an important predictor in the management of osteosarcoma patients [3, 44]. Finally, we followed a methodological and statistical standard in the derivation of the prognostic score and radiomics model development [15, 45]. All factors included in the model were pre-selected based on previous clinical evidence, clinical experience, and statistical significance. The predictors included in our model are routinely available baseline MRI and clinical data and are supported by solid theoretical foundations, including external validation.

This study has several limitations. First, its retrospective design and long inclusion period resulted in heterogeneity in MRI protocols, vendors, and field strengths, which may have influenced radiomic features but reflect real-world practice. Fat-suppressed T2-weighted imaging was performed using either spectral fat suppression or STIR; although STIR was used in a small proportion of cases, subgroup and external validation analyses (using only fat-suppressed T2-weighted imaging) demonstrated preserved performance. Results were also consistent with prior studies using T2-weighted alone [10] or combined T2-weighted/STIR sequences [14]. Second, differences between the internal and external cohorts may have introduced confounding; however, model performance remained stable across cohorts. Third, radiomic analysis was limited to baseline T2-weighted imaging at a single time point, without advanced sequences (*e.g*., diffusion-weighted imaging, dynamic contrast-enhanced studies) [46, 47] or longitudinal imaging [48], which may improve performance but are not routinely available and may delay treatment adaptation [8, 21]. Fourth, PET, CT, and laboratory biomarkers (*e.g*., alkaline phosphatase, lactate dehydrogenase) were not included, as they are optional, inconsistently performed, or add limited independent prognostic value beyond established clinical and conventional imaging factors [7]. Fifth, histologic assessment and segmentation were performed or confirmed by a single specialist at each institution, precluding formal interobserver analysis; however, discrepancies in segmentation were resolved by consensus. Finally, although the sample size was modest, it is comparable to the largest published MRI radiomics cohorts in osteosarcoma [15]. However, prospective multicenter validation and cost-effectiveness analyses are warranted to further establish clinical utility.

In conclusion, this study developed and externally validated a clinically oriented radiomics model integrating baseline T2-weighted MRI features with routinely available clinical-imaging parameters to predict chemoresistance in high-grade osteosarcoma. The model demonstrated consistent performance across internal and external cohorts, supporting its robustness and potential generalizability. By relying solely on standard-of-care data, this approach is readily applicable in routine practice. Early identification of patients at risk for poor response to neoadjuvant chemotherapy may facilitate risk-adapted management, including closer monitoring, altered chemotherapy regimens, or earlier surgical intervention. Prospective multicenter validation is required to confirm clinical utility and support implementation.

## Supplementary information

**Additional File:** **Table S1** Parameters used for the sequences at our institution for internal dataset (MRI 1.5-T Signa Excite HD or MRI 1.5-T Signa HDxt, GE Healthcare, Best, Netherlands). **Table S2** Parameters used for the sequences at our institution for internal dataset (MRI 3-T Signa Pioneer GE Healthcare, Best, Netherlands). **Table S3** Parameters used in the MRI sequences for internal dataset (1.5T Magnetom, Siemens Healthcare, Erlangen, Germany). **Table S4** Parameters used in the MRI sequences for external validation dataset (1.5-T Ingenia, Philips Healthcare, New York, NY, USA). **Table S5** The top 20 radiomics features ranked by their weight obtained from a logistic regression model with L1 regularization.

## Data Availability

The data used are confidential. The datasets generated or analyzed during this study are available from the corresponding author on reasonable request, as well as source codes and the final model.

## Abbreviations

AUROC: Area under the receiver operating characteristic curve (AUROC)
BMI: Body mass index
CT: Computed tomography
GLCM: Gray-level co-occurrence matrix
GLDM: Gray-level dependence matrix
GLRLM: Gray-level run-length matrix
GLSZM: Gray-level size-zone matrix
kNN: k-nearest neighbor
MRI: Magnetic resonance imaging
STIR: Short tau inversion recovery
SVM: Support vector machine

## References

[1] Jawad MU, Cheung MC, Clarke J, Koniaris LG, Scully SP (2011) Osteosarcoma: improvement in survival limited to high-grade patients only. J Cancer Res Clin Oncol 137:597–607. 10.1007/s00432-010-0923-7 20514491 10.1007/s00432-010-0923-7 PMC11827950

[2] Mirabello L, Troisi RJ, Savage SA (2009) Osteosarcoma incidence and survival rates from 1973 to 2004: data from the Surveillance, Epidemiology, and End Results Program. Cancer 115:1531–1543. 10.1002/cncr.24121 19197972 10.1002/cncr.24121 PMC2813207

[3] Bielack SS, Kempf-Bielack B, Delling G et al (2002) Prognostic factors in high-grade osteosarcoma of the extremities or trunk: an analysis of 1,702 patients treated on neoadjuvant cooperative osteosarcoma study group protocols. J Clin Oncol 20:776–790. 10.1200/jco.2002.20.3.776 11821461 10.1200/JCO.2002.20.3.776

[4] Hendershot E, Pappo A, Malkin D, Sung L (2006) Tumor necrosis in pediatric osteosarcoma: impact of modern therapies. J Pediatr Oncol Nurs 23:176–181. 10.1177/1043454206289786 16766682 10.1177/1043454206289786

[5] Kubo T, Furuta T, Johan MP, Adachi N, Ochi M (2016) Percent slope analysis of dynamic magnetic resonance imaging for assessment of chemotherapy response of osteosarcoma or Ewing sarcoma: systematic review and meta-analysis. Skeletal Radiol 45:1235–1242. 10.1007/s00256-016-2410-y 27229874 10.1007/s00256-016-2410-y

[6] Laux CJ, Berzaczy G, Weber M et al (2015) Tumour response of osteosarcoma to neoadjuvant chemotherapy evaluated by magnetic resonance imaging as prognostic factor for outcome. Int Orthop 39:97–104. 10.1007/s00264-014-2606-5 25432323 10.1007/s00264-014-2606-5

[7] Biermann JS, Hirbe A, Ahlawat S et al (2025) Bone cancer, version 2.2025, NCCN clinical practice guidelines in oncology. J Natl Compr Canc Netw. 10.6004/jnccn.2025.0017 10.6004/jnccn.2025.0017 40203873

[8] Nguyen JC, Baghdadi S, Pogoriler J, Guariento A, Rajapakse CS, Arkader A (2022) Pediatric osteosarcoma: correlation of imaging findings with histopathologic features, treatment, and outcome. Radiographics 42:1196–1213. 10.1148/rg.210171 35594197 10.1148/rg.210171

[9] Kanthawang T, Pattamapaspong N, Settakorn J et al (2025) Development and validation of a predictive score for chemoresistance in high-grade osteosarcoma at baseline. Front Med 12:1588302. 10.3389/fmed.2025.1588302 10.3389/fmed.2025.1588302 PMC12271180 40687707

[10] White LM, Atinga A, Naraghi AM et al (2023) T2-weighted MRI radiomics in high-grade intramedullary osteosarcoma: predictive accuracy in assessing histologic response to chemotherapy, overall survival, and disease-free survival. Skeletal Radiol 52:553–564. 10.1007/s00256-022-04098-2 35778618 10.1007/s00256-022-04098-2

[11] Prudowsky ZD, Yustein JT (2020) Recent insights into therapy resistance in osteosarcoma. Cancers. 10.3390/cancers13010083 10.3390/cancers13010083 PMC7795058 33396725

[12] Chen H, Zhang X, Wang X et al (2021) MRI-based radiomics signature for pretreatment prediction of pathological response to neoadjuvant chemotherapy in osteosarcoma: a multicenter study. Eur Radiol 31:7913–7924. 10.1007/s00330-021-07748-6 33825032 10.1007/s00330-021-07748-6

[13] Zhang L, Ge Y, Gao Q et al (2021) Machine learning-based radiomics nomogram with dynamic contrast-enhanced MRI of the osteosarcoma for evaluation of efficacy of neoadjuvant chemotherapy. Front Oncol 11:758921. 10.3389/fonc.2021.758921 34868973 10.3389/fonc.2021.758921 PMC8634262

[14] Zhong J, Zhang C, Hu Y et al (2022) Automated prediction of the neoadjuvant chemotherapy response in osteosarcoma with deep learning and an MRI-based radiomics nomogram. Eur Radiol 32:6196–6206. 10.1007/s00330-022-08735-1 35364712 10.1007/s00330-022-08735-1

[15] Zhong J, Hu Y, Zhang G et al (2022) An updated systematic review of radiomics in osteosarcoma: utilizing CLAIM to adapt the increasing trend of deep learning application in radiomics. Insights Imaging 13:138. 10.1186/s13244-022-01277-6 35986808 10.1186/s13244-022-01277-6 PMC9392674

[16] Holscher HC, Bloem JL, van der Woude HJ et al (1995) Can MRI predict the histopathological response in patients with osteosarcoma after the first cycle of chemotherapy? Clin Radiol 50:384–390. 10.1016/s0009-9260(05)83135-6 7789022 10.1016/s0009-9260(05)83135-6

[17] Zhang B, Zhang Y, Li R, Li J, Lu X, Zhang Y (2020) The efficacy and safety comparison of first-line chemotherapeutic agents (high-dose methotrexate, doxorubicin, cisplatin, and ifosfamide) for osteosarcoma: a network meta-analysis. J Orthop Surg Res 15:51. 10.1186/s13018-020-1576-0 32054494 10.1186/s13018-020-1576-0 PMC7020590

[18] Kocak B, Baessler B, Bakas S et al (2023) CheckList for EvaluAtion of Radiomics research (CLEAR): a step-by-step reporting guideline for authors and reviewers endorsed by ESR and EuSoMII. Insights Imaging 14:75. 10.1186/s13244-023-01415-8 37142815 10.1186/s13244-023-01415-8 PMC10160267

[19] Jawad MU, Scully SP (2010) In brief: Classifications in brief: Enneking classification: benign and malignant tumors of the musculoskeletal system. Clin Orthop Relat Res 468:2000–2002. 10.1007/s11999-010-1315-7 20333492 10.1007/s11999-010-1315-7 PMC2882012

[20] Fayad LM, Jacobs MA, Wang X, Carrino JA, Bluemke DA (2012) Musculoskeletal tumors: how to use anatomic, functional, and metabolic MR techniques. Radiology 265:340–356. 10.1148/radiol.12111740 23093707 10.1148/radiol.12111740 PMC3480818

[21] Saifuddin A, Sharif B, Gerrand C, Whelan J (2019) The current status of MRI in the pre-operative assessment of intramedullary conventional appendicular osteosarcoma. Skeletal Radiol 48:503–516. 10.1007/s00256-018-3079-1 30288560 10.1007/s00256-018-3079-1

[22] Enneking WF, Spanier SS, Goodman MA (1980) Current concepts review. The surgical staging of musculoskeletal sarcoma. J Bone Jt Surg Am 62:1027–1030 7000786

[23] Kanthawang T, Wudhikulprapan W, Phinyo P et al (2024) Can conventional magnetic resonance imaging at presentation predict chemoresistance in osteosarcoma? Br J Radiol 97:451–461. 10.1093/bjr/tqad047 38308035 10.1093/bjr/tqad047

[24] Fujifilm SYNAPSE 3D. Available via https://synapse-emea.fujifilm.com/synapse-3d.html. (Accessed 23 Mar 2026)

[25] Picci P, Bacci G, Campanacci M et al (1985) Histologic evaluation of necrosis in osteosarcoma induced by chemotherapy. Regional mapping of viable and nonviable tumor. Cancer 56:1515–1521. https://doi.org/10.1002/1097-0142(19851001)56:7<1515::aid-cncr2820560707>3.0.co;2-6 3861228 10.1002/1097-0142(19851001)56:7<1515::aid-cncr2820560707>3.0.co;2-6

[26] Haralick RM, Shanmugam K, Dinstein I (1973) Textural features for image classification. IEEE Trans Syst Man Cybern 3:610–621. 10.1109/TSMC.1973.4309314

[27] Sun C, Wee WG (1983) Neighboring gray level dependence matrix for texture classification. Comput Vis Graph Image Process 23:341–352. 10.1016/0734-189X(83)90032-4

[28] Galloway MM (1975) Texture analysis using gray level run lengths. Comput Graph Image Process 4:172–179. 10.1016/S0146-664X(75)80008-6

[29] Thibault G, Fertil B, Navarro CL et al (2009) Texture indexes and gray level size zone matrix: application to cell nuclei classification. In: 10th International Conference on Pattern Recognition and Information Processing. PRIP 2009, Minsk, Belarus, pp. 140–145. https://hal.science/hal-01499715

[30] Amadasun M, King R (1989) Textural features corresponding to textural properties. IEEE Trans Syst Man Cybern 19:1264–1274. 10.1109/21.44046

[31] Van Griethuysen JJM, Fedorov A, Parmar C et al (2017) Computational radiomics system to decode the radiographic phenotype. Cancer Res 77:e104–e107. 10.1158/0008-5472.CAN-17-0339 29092951 10.1158/0008-5472.CAN-17-0339 PMC5672828

[32] Lambin P, Leijenaar RTH, Deist TM et al (2017) Radiomics: the bridge between medical imaging and personalized medicine. Nat Rev Clin Oncol 14:749–762. 10.1038/nrclinonc.2017.141 28975929 10.1038/nrclinonc.2017.141

[33] Altmann A, Toloşi L, Sander O, Lengauer T (2010) Permutation importance: a corrected feature importance measure. Bioinformatics 26:1340–1347. 10.1093/bioinformatics/btq134 20385727 10.1093/bioinformatics/btq134

[34] Jeong SY, Kim W, Byun BH et al (2019) Prediction of chemotherapy response of osteosarcoma using baseline ^18^F-FDG textural features machine learning approaches with PCA. Contrast Media Mol Imaging 2019:3515080. 10.1155/2019/3515080 31427908 10.1155/2019/3515080 PMC6681577

[35] Bouhamama A, Leporq B, Khaled W et al (2022) Prediction of histologic neoadjuvant chemotherapy response in osteosarcoma using pretherapeutic MRI radiomics. Radiol Imaging Cancer 4:e210107. 10.1148/rycan.210107 36178349 10.1148/rycan.210107 PMC9530773

[36] Roudi R, Pisani LJ, Pisani F, Liang T, Daldrup-Link HE (2024) Reproducibility and repeatability of quantitative T2 and T2* mapping of osteosarcomas in a mouse model. Eur Radiol Exp 8:74. 10.1186/s41747-024-00467-9 38872042 10.1186/s41747-024-00467-9 PMC11176138

[37] Withers HR, Lee SP (2006) Modeling growth kinetics and statistical distribution of oligometastases. Semin Radiat Oncol 16:111–119. 10.1016/j.semradonc.2005.12.006 16564446 10.1016/j.semradonc.2005.12.006

[38] Pervaiz S (2002) Anti-cancer drugs of today and tomorrow: are we close to making the turn from treating to curing cancer? Curr Pharm Des 8:1723–1734. 10.2174/1381612023394025 12171544 10.2174/1381612023394025

[39] Inarejos Clemente EJ, Navarro OM, Navallas M et al (2022) Multiparametric MRI evaluation of bone sarcomas in children. Insights Imaging 13:33. 10.1186/s13244-022-01177-9 35229206 10.1186/s13244-022-01177-9 PMC8885969

[40] Uhl M, Saueressig U, van Buiren M et al (2006) Osteosarcoma: preliminary results of *in vivo* assessment of tumor necrosis after chemotherapy with diffusion- and perfusion-weighted magnetic resonance imaging. Invest Radiol 41:618–623. 10.1097/01.rli.0000225398.17315.68 16829744 10.1097/01.rli.0000225398.17315.68

[41] Beltran J, Simon DC, Katz W, Weis LD (1987) Increased MR signal intensity in skeletal muscle adjacent to malignant tumors: pathologic correlation and clinical relevance. Radiology 162:251–255. 10.1148/radiology.162.1.3786772 3786772 10.1148/radiology.162.1.3786772

[42] Holscher HC, Bloem JL, Vanel D et al (1992) Osteosarcoma: chemotherapy-induced changes at MR imaging. Radiology 182:839–844. 10.1148/radiology.182.3.1535905 1535905 10.1148/radiology.182.3.1535905

[43] Masrouha KZ, Musallam KM, Samra AB et al (2012) Correlation of non-mass-like abnormal MR signal intensity with pathological findings surrounding pediatric osteosarcoma and Ewing’s sarcoma. Skeletal Radiol 41:1453–1461. 10.1007/s00256-012-1383-8 22406919 10.1007/s00256-012-1383-8

[44] Xin S, Wei G (2020) Prognostic factors in osteosarcoma: a study level meta-analysis and systematic review of current practice. J Bone Oncol 21:100281. 10.1016/j.jbo.2020.100281 32140401 10.1016/j.jbo.2020.100281 PMC7047183

[45] Kocak B, Akinci D’Antonoli T, Mercaldo N et al (2024) METhodological RadiomICs Score (METRICS): a quality scoring tool for radiomics research endorsed by EuSoMII. Insights Imaging 15:8. 10.1186/s13244-023-01572-w 38228979 10.1186/s13244-023-01572-w PMC10792137

[46] Bajpai J, Gamnagatti S, Kumar R et al (2011) Role of MRI in osteosarcoma for evaluation and prediction of chemotherapy response: correlation with histological necrosis. Pediatr Radiol 41:441–450. 10.1007/s00247-010-1876-3 20978754 10.1007/s00247-010-1876-3

[47] Zhao S, Su Y, Duan J et al (2019) Radiomics signature extracted from diffusion-weighted magnetic resonance imaging predicts outcomes in osteosarcoma. J Bone Oncol 19:100263. 10.1016/j.jbo.2019.100263 31667064 10.1016/j.jbo.2019.100263 PMC6812010

[48] Lin P, Yang PF, Chen S et al (2020) A Delta-radiomics model for preoperative evaluation of neoadjuvant chemotherapy response in high-grade osteosarcoma. Cancer Imaging 20:7. 10.1186/s40644-019-0283-8 31937372 10.1186/s40644-019-0283-8 PMC6958668

[49] Jolliffe IT (2002) Principal component analysis, 2nd edn. Springer, New York

[50] Lorensen WE, Cline HE (1987) Marching cubes: a high resolution 3D surface construction algorithm. In: Proceedings of the 14th annual conference on computer graphics and interactive techniques. Association for Computing Machinery, pp 163–169. 10.1145/37401.37422

[51] Dhawan AP (2008) Wavelet transform and its applications in medical image analysis. Principles and advanced methods in medical imaging and image analysis. World Scientific, pp 437–454. 10.1142/9789812814807_0018

