# Supplementary material for: Predicting resistance to neoadjuvant chemotherapy in osteosarcoma using machine learning with clinical data and T2-weighted MRI radiomics
Source: Eur Radiol Exp. 2026 May 20;10:69. doi: 10.1186/s41747-026-00732-z (PMC13191004; doi:10.1186/s41747-026-00732-z)
Supplement: Supplementary file 1 — Additional File: Table S1 Parameters used for the sequences at our institution for internal dataset (MRI 1.5-T Signa Excite HD or MRI 1.5-T Signa HDxt, GE Healthcare, Best, Netherlands). Table S2 Parameters used for the sequences at our institution for internal dataset (MRI 3-T Signa Pioneer GE Healthcare, Best, Netherlands). Table S3 Parameters used in the MRI sequences for internal dataset (1.5T Magnetom, Siemens Healthcare, Erlangen, Germany). Table S4 Parameters used in the MRI sequences for external validation dataset (1.5-T Ingenia, Philips Healthcare, New York, NY, USA). Table S5 The top 20 radiomics features ranked by their weight obtained from a logistic regression model with L1 regularization. [file 41747_2026_732_MOESM1_ESM.pdf]

Predicting resistance to neoadjuvant chemotherapy in osteosarcoma using machine learning with clinical data and T2-weighted MRI radiomics

ELECTRONIC SUPPLEMENTARY MATERIAL

Table S1 Parameters used for the sequences at our institution for internal dataset (MRI 1.5-T Signa Excite HD or MRI 1.5-T Signa HDxt, GE Healthcare, Best, Netherlands)

Sequences	TR (ms)	TE (ms)	ST (mm)	Spacing (mm)	NEX	Echo train length	Matrix
Axial T1-weighted with and without FS*	600–800	9–11	4–5	4.50–6.50	1	1	160–192 × 320–380
Axial T2-weighted FS*	3,400–4,200	0–100	4–5	4.50–6.50	1	1	160–220 × 256–380
Axial 2D MERGE*,**	700	5	5–6	6–7	1	1	192 × 320
Sagittal T1*	600–800	11	4–5	4.50–6.50	1	1	160–192 × 320–380
Sagittal T2-weighted FS*	3,400–4,200	0–100	4–5	4.50–6.50	1	1	160–220 × 256–380
Coronal T1*	600–800	11	4–5	4.50–6.50	1	1	160–192 × 320–380
Coronal T2-weighted FS*	3,400–4200	0–100	4–5	4.50–6.50	1	1	160–220 × 256–380
Three views of post-contrast T1-weighted FS*,***	600–800	11	4–5	4.50–6.50	1	1	160–192 × 320–380
Coronal T1-weighted of the affected extremity*	620–1,160	9–10	4–5	4.50–5.50	1	1	192 × 320

*Field of view (FOV) depends on tumor size and site. **Flip angle = 20°. *** The subtraction technique was routinely applied in the axial view. *FS* Fat suppression, *NEX* Number of excitations, *ST* Slice thickness, *TE*, echo time; *TR* Repetition time.

Table S2 Parameters used for the sequences at our institution for internal dataset (MRI 3-T Signa Pioneer GE Healthcare, Best, Netherlands)

Sequences	TR (ms)	TE (ms)	ST (mm)	Spacing (mm)	NEX	Echo train length	Matrix
Axial T1-weighted with and without FS*	500–850	9–11	4–6	0.4–1	1	3–5	320–420 × 224–320
Axial T2-weighted FS*	3000–6000	60–102	4–6	0.4–1	1	16–30	320–420 × 224–320
Axial 2D MERGE*,**	400–700	8	4–6	6–7	1	–	352 × 240
Sagittal T1*	500–850	9–11	4–5	0.4–0.5	1	3–5	452–640 × 256–320
Sagittal T2-weighted FS*	3000–6000	60–102	4–5	0.4–0.5	1	16–30	384–512 × 256–320
Coronal T1*	500–850	9–11	4–5	0.4–0.5	1	3–5	452–640 × 256–320
Coronal T2-weighted FS*	3000–6000	60–102	4–5	0.4–0.5	1	16–30	384–512 × 256–320
Three views of post-contrast T1-weighted FS*,***	500–850	9–11	4–6	0.4–1	1	3–5	320–512 × 224–320
Coronal T1-weighted of the affected extremity*	500–850	9–11	4–5	0.4–0.5	1	3–5	320–512 × 224–320

*Field of view (FOV) depends on tumor size and site. **Flip angle = 20°. *** The subtraction technique was routinely applied in the axial view. *FS* Fat suppression, *NEX* Number of excitations, *ST* Slice thickness, *TE*, echo time; *TR* Repetition time.

Table S3 Parameters used in the MRI sequences for internal dataset (1.5T Magnetom, Siemens Healthcare, Erlangen, Germany)

Sequences	TR (ms)	TE (ms)	ST (mm)	Spacing (mm)	NEX	Echo train length	Matrix
Axial T1-weighted*	660–700	9.0–9.5	4–5	4.50–6.50	1	1	192 × 320
Axial STIR*,**	3,900–4,100	30–45	4–5	4.00–5.50	1	1	192 × 256
Sagittal T1*	660–700	9.0–9.5	4–5	4.50–6.50	1	1	192 × 320
Sagittal STIR*,**	3900–4,100	30–45	4–5	4.00–5.50	1	1	192 × 256
Coronal T1*	660–700	9.0–9.5	4–5	4.50–6.50	1	1	192 × 320
Coronal STIR*,**	3,900–4100	30–45	4–5	4.00–5.50	1	1	192 × 256
Three views of post-contrast T1-weighted FS	700–760	9.00–9.60	4–5	4.00–4.50	1	2	192 × 320
Coronal T1-weighted of the affected extremity*	660–700	9.0–9.5	4–5	4.50–6.50	1	1	192 × 320
Coronal STIR of the affected extremity*,**	3,900–4,100	30–45	4–5	4.00–5.50	1	1	192 × 256

*Field of view (FOV) depends on tumor size and site. **Time to inversion (TI) = 160 ms. *FS* Fat suppression, *NEX* Number of excitations, *TE* Echo time, *TR* Repetition time, *ST* Slice thickness, *STIR* Short tau inversion recovery.

Table S4 Parameters used in the MRI sequences for external validation dataset (1.5-T Ingenia, Philips Healthcare, New York, NY, USA)

Sequences	TR (ms)	TE (ms)	ST (mm)	Spacing (mm)	NEX	Echo train length	Matrix
Axial T1-weighted*	500–800	8–15	3–8	3–9.6	1	1	228–384 × 210–380
Axial T2-weighted*	2,500–4,700	70–80	3–8	3–9.6	1	1	227–384 × 146–380
T2-weighted FFE*, **	300–500	10–23	3–8	3–9.6	1	1	129–320 × 148–235
Coronal T1*	480–800	10–15	3–4	3.3–4.4	1	1	224–360 × 232–311
Coronal T2-weighted FS*	2,500-4,600	70–80	3–4	3.3–4	1–2	1	237–246 × 312–512
Sagittal T2-weighted FS*	2,500–4,200	60–80	3	3–3.3	1	1	159–308 × 188–356
Post contrast T1-weighted FS*	500–800	10–15	3–8	3–9.6	1–2	1	209–320 × 190–418

*Field of view (FOV) depends on tumor size and site. **Flip angle = 20°. *FS* Fat suppression, *NEX* Number of excitations, *ST* Slice thickness, *TE* Echo time, *TR* Repetition time.

Table S5 The top 20 radiomics features ranked by their weight obtained from a logistic regression model with L1 regularization

Number	Feature	Coefficient (weight)	Odds ratio	Absolute coefficient	*p*-value
1	original_shape_MinorAxisLength_Tomo	0.000636	1.000636	0.000636	0.007223
2	original_shape_Maximum2DDiameterColumn_Peri	0.004786	1.004798	0.004786	0.011077
3	original_gldm_LargeDependenceLowGrayLevelEmphasis_Peri	-0.003822	0.996185	0.003822	0.012914
4	original_glrlm_RunLengthNonUniformity_Tomo	0.0000004	1.0000004	0.0000004	0.013466
5	wavelet-LLH_glrlm_RunLengthNonUniformity_Tomo	0.000001	1.000001	0.000001	0.013627
6	original_shape_MeshVolume_Tomo	0.0000005	1.0000005	0.0000005	0.013647
7	wavelet-LLL_glrlm_RunLengthNonUniformity_Tomo	0.0000001	1.0000001	0.0000001	0.013867
8	wavelet_HLH_glcm_Idmn_Tomo	-0.00000002	0.9999999	0.00000002	0.014423
9	original_shape_VoxelVolume_Tomo	0.0000006	1.0000006	0.0000006	0.017619
10	wavelet-HHH_gldm_DependenceNonUniformity_Tomo	0.0000043	1.0000043	0.0000043	0.019000
11	wavelet-HLH_glcm_Idn_Tomo	0.001601	1.001603	0.001602	0.019679
12	log-sigma-1-0-mm-3D_glrlm_RunLengthNonUniformity_Tomo	-0.00000046	0.9999995	0.00000046	0.021133
13	log-sigma-3-0-mm-3D_glrlm_RunLengthNonUniformity_Tomo	-0.00000050	0.9999995	0.00000050	0.022402
14	wavelet-HHL_gldm_DependenceNonUniformity_Tomo	0.00001109	1.0000111	0.00001109	0.024585
15	wavelet-LLL_glszm_SizeZoneNonUniformity_Tomo	0.0000009	1.0000009	0.0000009	0.024621
16	wavelet-LHL_glrlm_RunLengthNonUniformity_Tomo	-0.00000022	0.9999998	0.00000022	0.024786
17	wavelet-LHH_glrlm_RunLengthNonUniformity_Tomo	0.00000028	1.0000003	0.00000028	0.024863
18	wavelet-HLH_glcm_Idmn_Peri	-0.00205336	0.9979487	0.00205336	0.025241
19	wavelet-HLH_glrlm_RunLengthNonUniformity_Tomo	-0.00000138	0.9999986	0.00000138	0.026627
20	original_shape_SurfaceArea_Tomo	0.0000007	1.0000007	0.0000007	0.026686
